# Analysis of the Dilemma of Promoting Circular Logistics Packaging in China: A Stochastic Evolutionary Game-Based Approach

**DOI:** 10.3390/ijerph19127363

**Published:** 2022-06-15

**Authors:** Xinyang Xu, Yang Yang

**Affiliations:** Faculty of Transportation Engineering, Kunming University of Science and Technology, 727 Jingming South Road, Kunming 650500, China; xuxinyang@stu.kust.edu.cn

**Keywords:** circular packaging, stochastic evolutionary game, regulatory policy, recycling incentives, information disclosure

## Abstract

The environmental pollution caused by logistics packaging in China has attracted increasing attention in recent years, and circular packaging is considered an effective means to solve the aforementioned problem. Therefore, this study considers the uncertainty of the external environment; constructs a stochastic game model of circular logistics-packaging promotion, which consists of environmental regulators, logistics enterprises, and consumers; collects data related to logistics packaging in China to describe the current circular-packaging promotion dilemma; and conducts a parameter-sensitivity analysis. The results show that (1) after a short period of fluctuation, the environmental regulator will lock in the “strong regulation” strategy, whereas logistics enterprises and consumers will quickly lock in the “no promotion” and “negative use” strategies. (2) The change in the initial probability will affect the rate of strategy evolution of the gaming system. (3) The “strong regulatory” strategy of environmental regulators and the increase in the number of circular-packaging cycles can help establish a logistics-recycling-packaging system. (4) The increase in recycling incentives can cause consumers to shift toward “active use” strategies, but this has accelerated the rate at which logistics companies lock into “no promotion” strategies. (5) The increase in the intensity of random interference will raise the fluctuation of the evolution of the game subject. For logistics enterprises, moderate random interference helps them evolve toward the “promotion” strategy.

## 1. Introduction

Recently, the logistics industry in e-commerce in China, driven by spectacular expansion, has significantly increased. From 2010 to 2020, logistics service enterprises’ business volume in China has increased from 2.34 billion pieces to 83.36 billion pieces, with an average annual compound growth rate of about 42.9%. In 2021, China’s business volume of logistics exceeded 100 billion pieces, ranking first in the world for eight consecutive years. China’s logistics parcel volume has exceeded that of the United States, Japan, Europe, and other well-developed economies, contributing to more than 50% to the world’s parcel growth, and has become a power source and stabilizer of the world’s postal industry. With the rapid development of the logistics industry, related environmental issues are of greater concern, and a large number of logistics-packaging materials that become waste will not only cause a waste of resources but also have a great environmental impact [1]. It is estimated that the logistics industry in China consumes more than 9 million tons of paper waste and about 1.8 million tons of plastic waste each year. If calculated by the industry standard of 0.2 kg per logistics packaging, the country’s logistics industry produced a total of more than 20 million tons of “sky” solid waste in 2021. According to the current growth rate of the logistics industry, its carbon emissions will exceed 32 million tons, and by 2025, China’s logistics-packaging-waste generation will reach 21.6 million tons, with treatment costs reaching more than CNY 3 billion and landfill disposal amounting to more than 1 million tons. Moreover, the existing logistics-packaging-waste classification and recycling system in China is not perfect, and a huge amount of unsealed logistics packaging is disposed and not effectively recycled.

By the end of 2020, the Chinese government has set the goal of “carbon peaking and carbon neutrality”. Following this target, the country aims to reach peak carbon dioxide (CO_2_) emissions by 2030 and achieve carbon neutrality by 2060. Thus, circular packaging is considered an effective means to solve logistics-packaging pollution [2,3,4]. The China Post Bureau formulated the “Logistics Express Packaging Management Measures” in 2021 to regulate the selection of logistics packaging and packaging operation standards. Logistics enterprises pay more attention to green packaging construction, and some companies have begun promoting circular logistics packaging, such as JingDong (JD) Logistics, which aims to accelerate the development and application of circular packaging and promote the simplicity of packaging; and ShunFeng (SF) Logistics, which uses a single material polypropylene honeycomb panel to independently develop a new circular logistics box. However, the road to the greening of logistics packaging is not smooth. The high cost of circular packaging [5,6], the incomplete recycling system [7,8], and the lack of rigid standards for logistics packaging [9,10] are the three major problems faced by the greening of logistics packaging. It has been investigated that most consumers will directly discard the logistics packaging after receiving the logistics express, causing environmental pollution [11,12]. Changes in regulatory strategies made by environmental regulators, such as policy makers, have a great impact on the promotion of circular logistics packaging [13]. Subsidy policy [14,15,16] is widely considered a method that can promote the development of green logistics; however, different subsidy methods and subsidy targets cause different effects [17]. The promotion of circular packaging requires logistics enterprises to pay additional costs, and when consumers do not have sufficient environmental awareness and do not actively use circular packaging, logistics enterprises will actually suffer great losses. Hence, recycling incentives for consumers are necessary [18,19,20]. Recycling incentives for consumers significantly increase the operating costs of logistics enterprises. Currently, the government’s environmental-regulation policy determines the choice of logistics enterprise strategy; therefore, the logistics-packaging-waste problem should look not only at logistics enterprises but also at environmental regulators and consumers. Collaborative construction of a logistics-recycling-packaging system will significantly achieve the reduction and recycling of logistics packaging.

In reality, the uncertain external environment [21] is also an important factor to consider for the promotion of circular logistics packaging, such as the interference of public opinion on the strategy choice of government [22] and logistics enterprises [23], the speculative psychology of logistics enterprises [24], and the irrational emotions of consumers [25]. Therefore, this study constructs a tripartite evolutionary game model under the stochastic disturbance environment, aiming to solve the following key problems: (1) What are the payoff matrix and replication dynamic equations for environmental regulators, logistics companies, and consumers in the logistics-circular-packaging promotion game? (2) Is there an equilibrium solution in the game system after adding random disturbances [26], and if so, what are the boundary conditions? (3) What is the current status of the promotion and recycling of circular logistics packaging in China? (4) What kind of regulatory strategy should be implemented by environmental regulators [27] in promoting circular logistics packaging? (5) What are the factors influencing logistics companies and consumers to promote and actively participate in green packaging? To answer these questions, this study focuses on the subsidies, penalties, and information-disclosure efforts [28] of environmental regulators; on recycling incentives given by logistics companies for consumers and the number of times that circular packaging can be recycled [29]; and on influencing factors such as the intensity of interference in a random environment. Gaussian white noise is introduced to build a stochastic game model for promoting circular logistics packaging, and the numerical approximation solution is solved using the Taylor expansion. The data related to circular logistics packaging are collected to analyze the current situation of promoting circular packaging in China, and a sensitivity analysis of the parameters is conducted using numerical simulation in an attempt to find a solution to the current dilemma of promoting circular packaging in China.

The primary research motivation of this study is to provide suggestions for the promotion of circular logistics packaging and the establishment of a recycling system, enriching the application of stochastic evolutionary game theory on green logistics.

The main innovation of this study includes the following aspects: firstly, we construct a dynamic game model for promoting circular logistics packaging, comprising environmental regulators, logistics enterprises, and consumers from the perspective of system engineering and chain. Secondly, the stochastic environment is considered to introduce Gaussian white noise to improve the replication dynamic equations of each subject, overcoming the shortcoming of the traditional evolutionary game, which can only have deterministic decisions, and is more in line with the real situation. Thirdly, the numerical boundary conditions in the stochastic environment are obtained based on real data to simulate the current logistics-recycling-packaging promotion dilemma in China. The next sections of this study are organized as follows: Section 2 reviews related studies, and Section 3 constructs a traditional evolutionary game model for environmental regulators, logistics firms, and consumers. Section 4 introduces Gaussian white noise to construct a stochastic evolutionary game model and obtains numerical boundary conditions. Section 5 collects data to describe the current logistics-recycling-packaging promotion dilemma in China and conducts sensitivity analysis. Section 6 discusses the conclusions and provides policy recommendations.

## 2. Literature Review

### 2.1. Green Logistics-Packaging Recycling and Management

Recently, logistics-packaging waste is growing rapidly; however, the current recycling and management of logistics-packaging waste is not ideal. In response to this problem, Zhang et al. [9] designed an intelligent green logistics-packaging and service system based on the Internet of Things and cloud-computing technology, and proved the efficiency and feasibility of the proposed green logistics-optimization method through case studies. Liu et al. [30] analyzed the current problems of logistics packaging from the perspectives of low-carbon, green, and humanized packaging and put forward the prospect of developing green logistics packaging. Zhang et al. [31] discussed the principles of green packaging from the connotation of green logistics-packaging management and proposed specific management strategies from both government and logistics enterprise levels. Kumar et al. [32] designed a logistics-packaging-recycling route-optimization model using a genetic algorithm that considers economic and environmental factors. Renugala et al. [33] used a literature-review approach to explore the influence of internal and external factors of choosing green technologies by logistics companies and to identify the important factors contributing to the sustainable development of the logistics industry among them. Laguir et al. [34] collected data from 232 French third-party logistics providers and concluded that green logistics packaging had a positive impact on the development of green supply chains. Sun et al. [35] used a traditional evolutionary game approach, considering the behavioral choices of the government, logistics companies, and consumers, and discussed the implementation of recyclable green packaging. From analyzing 561 questionnaires, Wu et al. [10] concluded that consumers are more willing to use green logistics packaging than ordinary packaging, and the government needs to play a central role in proposing solutions to the logistics-packaging-pollution problem. Wang et al. [36] quantified the external cost of packaging for logistics enterprises and analyzed the investment in green packaging technology by logistics enterprises, considering government incentives. Wandosell et al. [37] analyzed the literature using the Scopus database with the help of visualization tools and discovered that logistics companies and consumers have become increasingly aware of the contribution of green packaging toward sustainable development in recent years. Li [38] introduced the green ecological theory, combined with the existing green packaging, from the perspective of visual-image design that aesthetics and novelty are useful in promoting green packaging applications. Hao et al. [39] used principal factor analysis and analyzed consumers’ willingness to use green packaging using 781 questionnaires, and the results showed that most consumers lacked awareness on green packaging and valued the practicality of green packaging more than its appearance.

Most of the aforementioned studies only examine the green packaging-management problem from the perspective of one subject and do not consider the strategic interaction of multiple subjects under the application of recycling and green logistics packaging. Therefore, this study builds a stochastic evolutionary game model that consists of the government, logistics enterprises, and consumers, and conducts an in-depth study on the promotion of circular packaging and the establishment of the recycling system.

### 2.2. Stochastic Evolutionary Game

Smith and Price [40] first introduced the concepts of evolutionary games and evolutionary stable strategies, marking the birth of evolutionary game theory [41]. Taylor and Jonker [42] first introduced the concept of replication-factor dynamics when studying ecoevolutionary phenomena, which is another groundbreaking development in evolutionary game theory. However, the traditional evolutionary game theory can only study the strategy evolution of each subject in a deterministic state and cannot describe the uncertainty in reality. Therefore, it is necessary to introduce random disturbances [43] to judge the stability of stochastic evolution under uncertain environments [44]. Chen and Yeh [45] introduced a stochastic genetic variation and stochastic environmental disturbances into a dynamic evolutionary game, constructed a nonlinear stochastic biological system, and simulated a simple stochastic evolutionary model. Stochastic evolutionary games have now expanded from the original study of biological populations to a variety of fields. Liu et al. [26] constructed a tripartite $\mathrm{It}\hat{o}$ stochastic evolutionary game model to study the problem in e-waste recycling management and provided constructive suggestions for the government to better formulate and implement environmental regulations. Li et al. [46] applied a stochastic evolutionary game approach to study the knowledge-sharing behavior of the members in public–private-partnership supply chains, arguing that firms with strong knowledge power are more sensitive to parameter changes than firms with weak knowledge power. Zhao et al. [47] applied a stochastic evolutionary game approach to construct a technology choice model of software vendors and consumers for the technical standard setting of competing software technologies. Lv et al. [48] combined stochastic process and evolutionary game theory to reveal the quality differences of package tours in different information conditions, establishing a composite mechanism in solving the quality problem of package tours. Li et al. [49] conducted a stochastic evolutionary game analysis of electrical vehicle-charging-facility construction strategies, which was simulated using data from Shanghai city and considered the strategic behaviors of the government, enterprises, and consumers.

The introduction of random interference coefficients in the traditional evolutionary game theory can effectively reflect the uncertain environment in reality; therefore, this study uses a stochastic evolutionary game approach to study the promotion and recycling of recycling packaging, in order to try to find a solution to the current promotion dilemma.

## 3. Problem Formulation and Model Construction

### 3.1. Problem Description and Parameter Setting

The three evolutionary game subjects in the logistics recycling system, namely environmental regulators, logistics enterprises, and consumers, are considered “limited rationality”. The role of the environmental regulator is to develop policies to promote green packaging, and they have two strategies: “strong regulation” and “weak regulation”. Strong regulation refers to the introduction of hard standards for green packaging in logistics by environmental regulators, prohibiting logistics enterprises from using packaging that does not meet green technical standards, exposing noncompliant enterprises and penalizing logistics enterprises that violate the ban. Weak regulation means that environmental regulators only subsidize logistics enterprises and do not regulate them; however, to promote the development of green logistics industry, they will subsidize logistics enterprises that use circular packaging regardless of the intervention policy adopted by environmental regulators. Logistics enterprises have two strategies: “promotion” and “nonpromotion”. The promotion strategy means that logistics enterprises promote circular packaging, which requires extra costs but will be subsidized by the government; and the nonpromotion strategy means that logistics enterprises do not promote circular packaging, which does not require extra costs but may be punished by the government. The consumer-behavior strategy comprises “active use” and “negative use”. The active-use strategy means that consumers actively use and recycle circular packaging and they will be rewarded for recycling; however, they will pay for the time and physical cost of recycling. The negative trial strategy means that consumers do not use circular packaging. Based on the problem description, we made the following assumptions:

**Assumption** **1.** *The subject of the game is finite-rational and will make judgments based on limited knowledge and information. It is also due to limited knowledge and information that each player will adjust their own strategy and pursue their own interest maximization in the game process based on the feedback of the behavior of the other party. The probability that the environmental regulator chooses the “weak regulation” strategy is* x *, and the probability of choosing the “strong regulation” strategy is* 1−x(0≤x≤1) *. The probability that a logistics company chooses the “nonpromotion” strategy is* y *, and the probability of choosing the “promotion” strategy is* 1−y(0≤y≤1) *. The probability that a consumer chooses the “negative use” strategy is* z *, and the probability of choosing the “active use” strategy is* 1−z(0≤z≤1) *.*

**Assumption** **2.** *The basic benefit to the environmental regulator is*$P_{1}$*, and the additional regulatory cost when choosing a strong regulatory strategy is*M. *When logistics companies promote circular packaging, whatever strategy the environmental regulators will choose gives logistics companies subsidies*θB*, where*θ*represents the level of subsidy from environmental regulators and*B*is the subsidy amount.Environmental regulators develop uniform logistics green standards when choosing a “strong regulation” strategy.Logistics companies are penalized if they do not promote circular packaging; they are fined*αG*, where*α*is the penalty strength and*G*is the penalty amount. Logistics companies will also be exposed to a decline in brand reputation, bringing losses*$\beta H_{1}$*, where*β*is the intensity of information disclosure by environmental regulators and*$H_{1}$*is the reputational damage of a logistics company’s choice of “nonpromotion” strategy when information is made public, whereas the government will gain credibility under strong regulation as*S*. Likewise, if logistics companies choose to promote circular packaging, they will receive a corporate reputation enhancement due to the disclosure of information by regulatory authorities*$\beta H_{2}$*, where*$H_{2}$*is the reputational benefits of a logistics company’s choice of “promotion” strategy when information is made public.*

**Assumption** **3.** *The basic revenue of a logistics company is*$P_{2}$*. The cost of using common logistics packaging for logistics companies is*$C_{1}$*. Ordinary logistics packaging has excessive packaging, and the use of nondegradable materials will cause pollution to the social environment; therefore, the government needs to pay the environmental management costs*J*. The cost of using circular packaging for logistics companies is*$C_{2}$*, and the maximum number of cycles that can be recycled for a circular package is*k*. Therefore, the actual cost of circular packaging*$C_{r}$*is*$\frac{C_{2}}{k}$. E*refers to logistics companies that develop the cost of circular packaging. Logistics enterprises that promote circular packaging also need to invest an additional cost,*W*, to establish packaging recycling points, hire packaging recycling staff, and give reward*R*to consumers who actively use circular packaging.*

**Assumption** **4.** *The basic benefit to the consumer is* $P_{3}$ *. Consumers using circular packaging will receive a reward of* R *from logistics companies; however, the act of recycling packaging will consume time and physical cost* T *. When consumers choose the “active use” strategy and logistics companies choose the “nonpromotion” strategy, consumers will suffer a psychological loss,* I *, due to the inability to use recycled packaging and will change the logistics company in the next service. The potential loss for logistics companies is* A *, and the negative use of circular packaging by consumers will also impose environmental control costs on environmental regulators.*

Based on the aforementioned assumptions, the parameters and descriptions are shown in Table 1 and Table 2.

### 3.2. Payoff Matrix and Replicator Dynamics Equations

According to the assumptions in Table 1 and Table 2, we can obtain the payment matrix of environmental regulators, logistics companies, and consumers, as shown in Table 3.

The expected return of the environmental regulator choosing the “weak regulation” strategy is $U_{g}^{w}$, the expected return of the “strong regulation” strategy is $U_{g}^{s}$, and the average expected return of the environmental regulator is ${\bar{U}}_{g}$.

(1) $$\begin{array}{cc} U_{g}^{w} & =yz(P_{1}-J)+(1-y)z(P_{1}-J-\theta B)+y(1-z)(P_{1}-J)+(1-y)(1-z)(P_{1}-\theta B)\\ & =P_{1}+\theta B(y-1)+Jyz-J(y+z) \end{array}$$

(2) $$\begin{array}{cc} U_{g}^{s} & =yz(P_{1}+\alpha G+\beta S-M-J)+(1-y)z(P_{1}+\beta S-M-\theta B-J)+\\ & y(1-z)(P_{1}+\alpha G+\beta S-M-J)+(1-y)(1-z)(P_{1}+\beta S-M-\theta B)\\ & =P_{1}-M+\beta S+\theta B(y-1)+\alpha Gy+Jyz-J(y+z) \end{array}$$

(3) $${\bar{U}}_{g}=xU_{g}^{w}+(1-x)U_{g}^{s}.$$

The replication dynamic equation for the environmental regulator can be obtained as follows:

(4) $$\begin{array}{cc} F(x) & =\frac{dx}{dt}=x(U_{g}^{w}-{\bar{U}}_{g})=x(1-x)(U_{g}^{w}-U_{g}^{s})\\ & =x(1-x)(M-\beta S-\alpha Gy) \end{array}$$

Similarly, the expected return of logistics enterprises choosing the “nonpromotion” strategy is $U_{l}^{n}$, the expected return of logistics enterprises choosing the “promotion” strategy is $U_{l}^{i}$, and the average expected return of logistics enterprises is ${\bar{U}}_{l}$.

(5) $$\begin{array}{cc} U_{l}^{n} & =xz(P_{2}-C_{1})+(1-x)z(P_{2}-C_{1}-\alpha G-\beta H_{1})+\\ & x(1-z)(P_{2}-C_{1}-A)+(1-x)(1-z)(P_{2}-C_{1}-\alpha G-\beta H_{1}-A).\\ & =P_{2}-C_{1}+\alpha G(x-1)+\beta H_{1}(x-1)+A(z-1) \end{array}$$

(6) $$\begin{array}{cc} U_{l}^{i} & =xz(P_{2}-C_{r}-E-W+\theta B)+(1-x)z(P_{2}-C_{r}-E-W+\theta B+\beta H_{2})\\ & +x(1-z)(P_{2}-C_{r}-E-W+\theta B-R)+(1-x)(1-z)(P_{2}-C_{r}-E-W+\theta B+\beta H_{2}-R)\\ & =P_{2}-C_{r}-E+\theta B-W+\beta (H_{2}-H_{2}x)+R(z-1) \end{array}$$

(7) $${\bar{U}}_{l}=xU_{l}^{n}+(1-x)U_{l}^{i}.$$

The replication dynamic equation of the logistics enterprises can be obtained as follows:

(8) $$\begin{array}{cc} F(y) & =\frac{dy}{dt}=y(U_{l}^{n}-{\bar{U}}_{l})=y(1-y)(U_{l}^{n}-U_{l}^{i})\\ & =y(1-y)[C_{r}-C_{1}+E+R-\theta B+W-\alpha G-\beta (H_{1}+H_{2})-A+\\ & \left(\alpha G+\beta (H_{1}+H_{2}))x+(A-R)z\right] \end{array}$$

Similarly, the expected benefit of consumers choosing the “negative use” strategy is $U_{c}^{p}$, the expected benefit of consumers choosing the “positive use” strategy is $U_{c}^{a}$, and the average expected benefit of logistics companies is ${\bar{U}}_{c}$.

(9) $$U_{c}^{p}=xyP_{3}+(1-x)yP_{3}+(1-x)(1-y)P_{3}+(1-y)xP_{3}=P_{3}.$$

(10) $$\begin{array}{cc} U_{c}^{a} & =xy(P_{3}-I)+(1-x)y(P_{3}-I)+x(1-y)(P_{3}+R-T)+(1-x)(1-y)(P_{3}+R-T)\\ & =y(T-I-R)+P_{3}+R-T \end{array}$$

(11) $${\bar{U}}_{c}=xU_{c}^{p}+(1-x)U_{c}^{a}.$$

The replication dynamic equation of the logistics enterprises can be obtained as follows:

(12) $$\begin{array}{cc} F(z) & =\frac{dz}{dt}=z(U_{c}^{p}-{\bar{U}}_{c})=z(1-z)(U_{c}^{p}-U_{c}^{a})\\ & =z(1-z)[T-R-y(T-I-R)] \end{array}$$

## 4. Construction of the Stochastic Evolutionary Game Model

### 4.1. Stochastic Evolutionary Game Model

The evolutionary game overcomes the shortcomings of the traditional game theory of complete rationality; however, the decision of each subject is deterministic, which is not consistent with the actual situation of most games; that is, the decision of each game group will suffer from the interference of some non-negligible random factors. The multibody game of circular-packaging promotion has great uncertainty. Firstly, even if there are incentives and penalties, speculation driven by profit may still exist. Secondly, there will be significant changes in the intensity of regulation under the supervision of public opinion and fluctuations in the management and decision-making system. Finally, the current logistics-packaging-recycling system in China has not been established, and the current situation of recycling is rather chaotic. Therefore, it is necessary to consider the disturbance of stochastic perturbation to the game system.

Loren [50] introduced Gaussian distribution to build an evolutionary model de-scribed by stochastic difference equations, successfully transferring Gaussian distribution from biological-science applications to management-science applications. At the same time, referring to Li et al. [49] introduced Gaussian distribution to the multiagent stochastic game study of charging facility construction. Liu et al. [26] introduced Gaussian distribution to the multiagent stochastic game study of electronic waste recycling. We introduce Gaussian white noise in this study to characterize the stochastic disturbance of the game system and to improve the replicated dynamic equation, obtaining the following:

(13) dx(t)=(M−βS−αGy)x(t)[1−x(t)]dt+σx(t)[1−x(t)]dωt,

(14) $$\begin{array}{l} dy(t)=[C_{r}-C_{1}+E+R-\theta B+W-\alpha G-\beta (H_{1}+H_{2})-A\\ +(\alpha G+\beta (H_{1}+H_{2}))x+(A-R)z]y(t)[1-y(t)]dt+\sigma y(t))[1-y(t)]d\omega (t) \end{array},$$

(15) dz(t)=[T−R−y(T−I−R)]z(t)[1−z(t)]dt+σz(t)[1−z(t)]dω(t),

where ω(t) is the standard one-dimensional Brown motion. It is an irregular random rise and fall phenomenon, which can well-describe the effect of random disturbance factors. dω(t) denotes the Gaussian white noise, when t>0, the step size is h>0; its increment Δω(t)=ω(t+h)−ω(t) obeys normal distribution $N(0,\sqrt{h})$; σ indicates random interference intensity. Equations (13)–(15) are one-dimensional $\mathrm{It}\hat{o}$ stochastic differential equations, replicated dynamic equations for environmental regulators, logistics firms, and manufacturers subjected to stochastic perturbations, respectively.

### 4.2. Analysis of the Existence and Stability of Equilibrium Solutions

For Equations (13)–(15), when the initial game t=0, that is, x(0)=0, y(0)=0, z(0)=0, we have the following:

(16) (M−βS−αGy)×0+σx(t)[1−x(t)]dω(t)=0. 

(17) $$\begin{array}{l} {}[C_{r}-C_{1}+E+R-\theta B+W-\alpha G-\beta (H_{1}+H_{2})-A+(\alpha G+\\ \beta (H_{1}+H_{2}))x+(A-R)z]\times 0+\sigma y(t)[1-y(t)]d\omega (t)=0 \end{array}$$

(18) [T−R−y(T−I−R)]×0+σz(t)[1−z(t)]dω(t)=0. 

From Equations (16)–(18), we can see that ${d\omega (t)|}_{t=0}=\omega^{\prime }(t){dt|}_{t=0}=0$. The equation has a zero solution; that is, it shows that the gaming system will stay in that state in the absence of white noise interferences. Therefore, the zero solution is the equilibrium solution of the equation, discriminating the stability of game systems according to the stability discriminant theorem for stochastic differential equations. First, given a stochastic differential equation:

(19) $$dx(t)=f(t,x(t))dt+g(t,x(t))d\omega (t),x(t_{0})=x_{0}.$$

Let there exist a function V(t,x) with a positive constant and $c_{1}$ and $c_{2}$, such that $c_{1}{\left|x\right|}^{p}\leq V(t,x)\leq c_{2}{\left|x\right|}^{p},t\geq 0$.

(a) If a positive constant λ exists, such that LV(t,x)≤−λV(t,x),t≥0, then the zero solution of Equation (19) is an exponentially stable P-order moment, which holds $E|{x(t,x_{0})|}^{p}<\left(\frac{c_{2}}{c_{1}}\right){\left|x_{0}\right|}^{p}e^{-\lambda t},t\geq 0$.
(b) If there exists a positive constant λ, such that LV(t,x)≥−λV(t,x),t≥0, then the zero solution of Equation (19) with the P-order moment exponent is unstable and holds $E|{x(t,x_{0})|}^{p}\geq \left(\frac{c_{2}}{c_{1}}\right){\left|x_{0}\right|}^{p}e^{-\lambda t},t\geq 0$.

For Equations (13)–(15), taking $V_{t}(t,x)=x$, $V_{t}(t,y)=y$, $V_{t}(t,z)=z$, x∈[0,1], y∈[0,1], z∈[0,1], $c_{1}=c_{2}=1$, p=1, and λ=1, we have the following:

(20) LV(t,x)=f(t,x)=(M−βS−αGy)x. 

(21) $$\begin{array}{l} LV(t,y)=f(t,y)=[C_{r}-C_{1}+E+R-\theta B+W-\alpha G-\beta (H_{1}+H_{2})\\ -A+(\alpha G+\beta (H_{1}+H_{2}))x+(A-R)z]y \end{array}$$

(22) LV(t,z)=f(t,z)=[T−R−y(T−I−R)]z. 

If the zero-solution moments of Equations (13)–(15) are exponentially stable, it is necessary to satisfy the following:

(23) (M−βS−αGy)x≤−x

(24) $$[C_{r}-C_{1}+E+R-\theta B+W-\alpha G-\beta (H_{1}+H_{2})-A+(\alpha G+\beta (H_{1}+H_{2}))x+(A-R)z]y\leq -y$$

(25) [T−R−y(T−I−R)]z≤−z.

### 4.3. Taylor Expansion of the Evolution Equation

Because the nonlinear $It\overset{\wedge }{o}$ stochastic differential equation cannot be solved analytically directly, it needs to be solved using a stochastic Taylor expansion, when $t_{0}=0$, t∈[0,T], the interval [0,T] is divided into $0=t_{0}<t_{1}<t_{2}<\ldots <t_{N}=T$, the average step size $t_{n}=nh$, and n=1,2,3,…,N. Written as $x(t_{0})=x_{0}$, $y(t_{0})=y_{0}$, and $z(t_{0})=z_{0}$, we assume $x_{0},y_{0},z_{0}\in R$.

The format of Euler’s explicit forward numerical method is $x_{n+1}=x_{n}+hf(x_{n})+\Delta \omega_{n}g(x_{n})$. Now, Equations (13)–(15) are expanded as above to obtain the following:

(26) $$x_{n+1}=x_{n}+(M-\beta S-\alpha Gy)xh+\Delta \omega_{n}\sigma x(n).\;$$

(27) $$\begin{array}{l} y_{n+1}=y_{n}+[C_{r}-C_{1}+E+R-\theta B+W-\alpha G-\beta (H_{1}+H_{2})-A\\ +(\alpha G+\beta (H_{1}+H_{2}))x+(A-R)z]yh+\Delta \omega_{n}\sigma y(n) \end{array}$$

(28) $$z_{n+1}=z_{n}+[T-R-y(T-I-R)]zh+\Delta \omega_{n}\sigma z(n).\;$$

**Figure 1:**
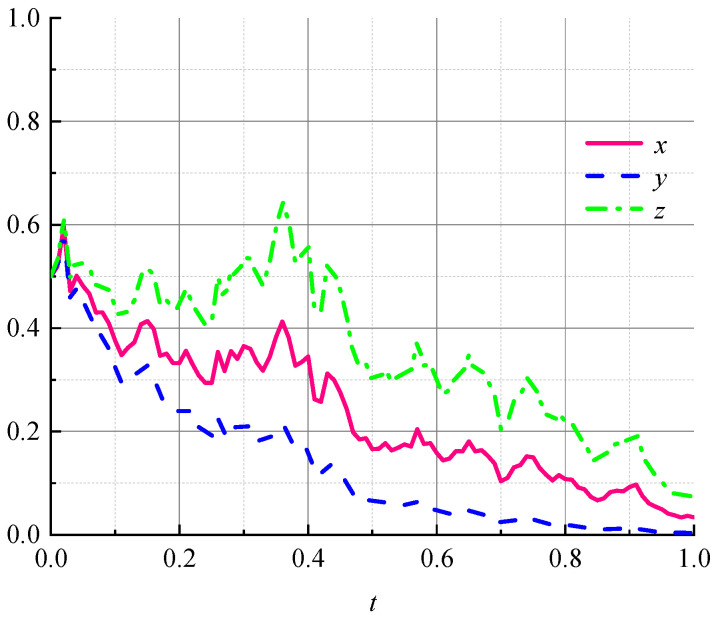
Tripartite dynamic evolutionary paths under stochastic disturbances.

We randomly set the values that satisfy the conditions (23)–(25). Let M=1, β=0.8, S=3, α=0.5, G=2, $C_{1}=1.5$, $C_{r}=0.5$, μ=0.8, E=2, R=2, θ=0.8, B=5, W=1, $H_{1}=1$, $H_{2}=1$, ε=0.5, A=2, T=0.5, I=0.5, σ=1, and x=y=z=0.5. The simulation results in Figure 1 demonstrate the validity of conditions (23)–(25), which means that the conditional boundaries of the subjects in the stochastic interference environment are given.

## 5. Simulation and Discussion

### 5.1. Data Collection

In this study, data related to logistics recycling packaging in China were collected to construct simulation values, as shown in Table 4. The specific instructions are as follows:

- JD Logistics has invested 300,000 “Qingliu” circular logistics boxes into society. The cost of this kind of logistics box is about CNY 15, and the number of cycles is about 20. The cost of an ordinary logistics box is about CNY 1; therefore, the use of ordinary logistics packaging cost $C_{1}$ is CNY 300,000. The cost of using circular logistics box $C_{r}$ is CNY 225,000.
- According to China’s Hainan Provincial Development and Reform Commission, each circular logistics box is subsidized by CNY 0.4. The subsidy θB of logistics companies obtained from environmental regulators is CNY 120,000. Assuming that the subsidy strength θ is 0.5, referring to Shanghai’s policy of penalizing enterprises with excessive carbon emissions, we can infer that logistics enterprises are fined αG CNY 100,000 for not using circular packaging, assuming that the penalty intensity α is 0.5 at this point.
- The cost of a green packaging recycling box is about CNY 500 per piece. According to “China Logistics Packaging Waste Generation Characteristics and Management Status Study Report,” it is estimated that 300,000 circular logistics boxes require about 100 recycling bins for their recovery. The personnel management cost of each recycling bin is about CNY 2000. Therefore, the additional cost W for logistics enterprises to promote circular packaging can be obtained as CNY 250,000. JD Logistics returns 20 “Jingdou” to consumers who take the initiative to recycle the packaging. “Jingdou” can be spent in JD Mall, and 20 “Jingdou” is about CNY 0.2. The incentive R given by logistics companies to consumers for using circular packaging can be obtained as CNY 60,000.
- The additional regulatory cost M is set at CNY 50,000 with reference to the public data of Shanghai Environmental Protection Bureau. Other parameters, which are more difficult to determine economically, are determined through expert interviews and visits to research organizations, where T is 0.6, I is 0.3, and $\beta H_{1}$ and $\beta H_{2}$ are both set to 2.
- The Chinese government is actively enacting various policies to reduce social carbon emissions and to achieve the goal of “carbon neutrality” and “carbon peaking”. Therefore, the probability of initial “weak regulation” by the environmental regulator *x* is 0.2. Most logistics companies are still in a wait-and-see situation, and the promotion of circular packaging is uncommon. Therefore, the initial “no promotion” probability *y* for logistics companies is 0.5. The survey shows that currently, only about 20% of consumers are actively recycling packaging. Therefore, the initial “negative use” probability *z* of the consumer is set to 0.8.

### 5.2. Simulation and Discussion

**Figure 2:**
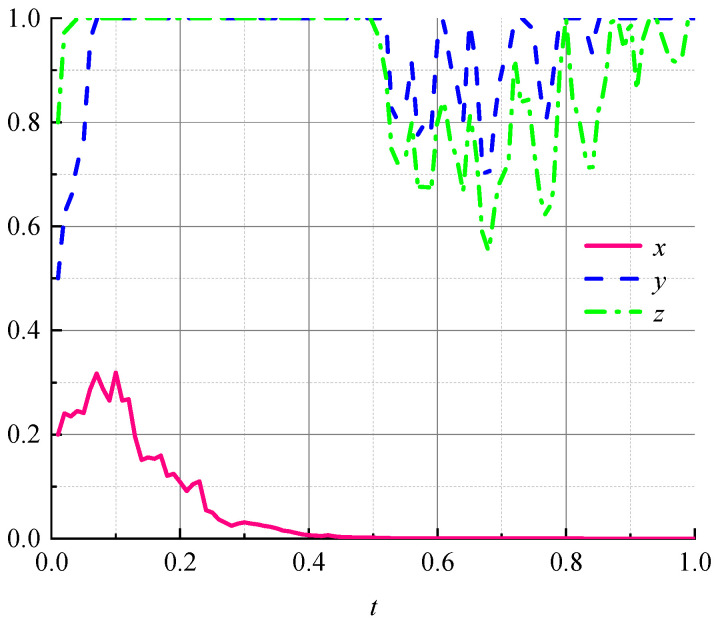
Evolution of the current status of the logistics-recycling system in China.

As shown in Table 4, we illustrated the current situation of promoting and using green packaging in Chinese logistics and used Matlab software (R2020a, MathWorks, (Natick, MA, USA)) to simulate the data in Table 4, and the simulation results are shown in Figure 2.

Figure 2 shows that the choice of “strong regulation” strategy at the beginning of the game is subject to fluctuations in the environment of random interference and briefly evolved in the direction of “weak regulation” over time. However, the ultimate choice of strategy for environmental regulators will stabilize, locking in the choice of the “strong regulation” strategy. Logistics companies and consumers are quick to lock in “nonpromotion” and “negative use” strategies in the early stages of the game. Due to the high initial probability of “negative use,” the rate of evolution of consumers to negative strategies is higher than the rate of evolution of logistics companies to nonpromotional strategies, and the volatility is less than the volatility of logistics companies to nonpromotional strategies. With environmental regulators locking in a “strong regulation” strategy, the strategic choices of logistics companies and consumers are once again shifting. At this time, the environmental regulatory policy under the “strong regulation” strategy of the environmental regulator encounters some effect, and logistics companies and consumers no longer firmly choose the “nonpromotion” and “negative use” strategies. At this time, consumers shift more than logistics companies; however, after a short period of fluctuation, the strategy choice of logistics companies will first stabilize and continue to lock in the “nonpromotion” strategy, and consumers will gradually lock in the “negative use” strategy after logistics companies lock in the “nonpromotion” strategy. It can be seen that the current status of the promotion and use of green logistics packaging in China is poor. Although environmental regulators are more determined to reduce carbon emissions and can steadily choose the “strong regulation” strategy under a random interference, logistics companies and consumers have difficulty locking in the “promotion” and “active use” strategies because of various complex reasons. Therefore, a sensitivity analysis of the important parameters in the stochastic game system will be conducted to investigate the influence of different parameters on the promotion of circular logistics packaging.

(1) Impact of initial probability changes on evolutionary results

**Figure 3:**
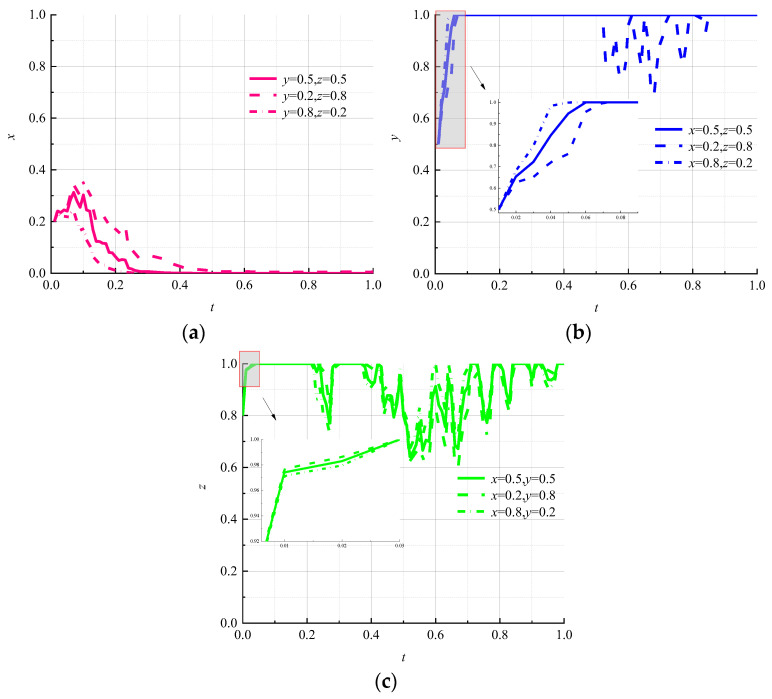
Evolutionary paths of each subject with different initial strategy-selection probabilities. (**a**) Environmental regulators; (**b**) logistics companies; (**c**) consumers.

We set 0.2 as the low initial probability, 0.5 as the medium initial probability, and 0.8 as the high initial probability to study the effect of random initial strategy combinations of two parties on the evolutionary outcome of the third party in a three-party game subject. The simulation results are shown in Figure 3.

The change in the probability of the initial strategy choice of logistics companies and consumers affects the rate of evolution of the environmental regulator to the “strong regulation” strategy, but does not change the strategy choice of the environmental regulator as shown in Figure 3a. When the initial probability of logistics companies choosing the “nonpromotion” strategy is 0.8 and the initial probability of consumers choosing the “negative use” strategy is 0.2, the rate of environmental regulators shifting to the “strong regulation” strategy is the fastest and the fluctuation of strategy selection is the least. Therefore, it can be seen that when the willingness of consumers to use circular packaging is high and the willingness of logistics enterprises to promote circular packaging is low, environmental regulators will quickly respond to the development of relevant logistics-packaging standards and will implement “strong regulation” policies to promote circular logistics packaging. In Figure 3b, it can be seen that when the combination of “weak regulation” and “negative use” strategies is (medium, medium) or (high, low) for the initial choice of environmental regulators and consumers, logistics companies are quick to lock in a “nonpromotion” strategy and remain stable under random interference. When the combination of “weakly regulated” and “negatively used” strategies is initially chosen by environmental regulators and consumers (low, high), the rate of evolution of logistics companies to “no promotion” strategy is slow and fluctuates. This indicates that the strategy choice of logistics companies tends to shift toward the “promotion” strategy under the government’s high-probability strong regulatory strategy, even though the willingness of consumers to use circular packaging is low. In Figure 3c, it can be seen that although consumers will quickly lock in the “negative use” strategy under the random-interference environment, their strategy choice will fluctuate for a long time and will not remain stable as time goes on. When the initial probability of environmental regulators choosing “weak regulation” is high and the initial probability of logistics companies choosing “nonpromotion” is low, consumers shift to the “negative use” strategy at the slowest and most volatile rate. This shows that consumers are more hesitant to actively use circular packaging even if the government does not implement sufficient environmental regulation policies, as the willingness of logistics companies to promote it increases.

(2) Impact of information disclosure intensity on evolutionary results

**Figure 4:**
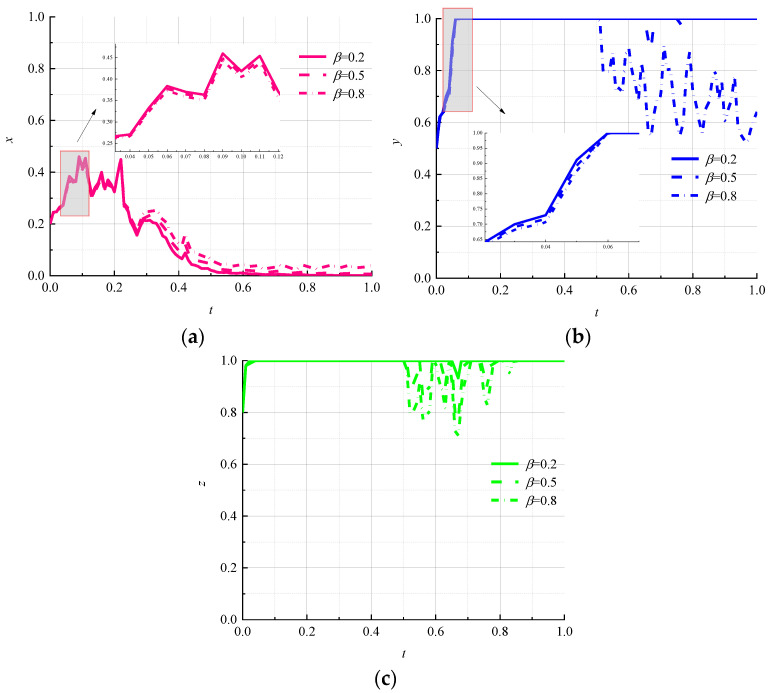
Evolutionary paths of each subject under different information-disclosure efforts. (**a**) Environmental regulators; (**b**) logistics companies; (**c**) consumers.

The strength of information disclosure affects the reputation of logistics companies and the credibility of environmental regulators. Let β be 0.2, 0.5, and 0.8, corresponding to low strength, medium strength, and high strength, respectively, to study the effect of different information-disclosure strengths on game outcomes. The simulation results are shown in Figure 4.

It can be seen in Figure 4a that the change in information disclosure will not change the choice of environmental regulators to the “strong regulation” strategy in a stochastic environment and will reduce the fluctuation of environmental regulators to lock in a “strong regulation” strategy in the early stage of the game; however, the shift of the government to a “strong regulation” strategy has been slowest under the high intensity of information disclosure. From Figure 4b, it can be seen that the rate of information disclosure is inversely proportional to the rate of evolution of logistics enterprises toward the “nonpromotion” strategy, and with the increase in information disclosure, logistics enterprises no longer lock in the “nonpromotion” strategy; hence, evolutionary paths show significant instability. It can be seen that the high intensity of information disclosure in the random-interference environment is conducive to the promotion of circular packaging by logistics enterprises. It can be seen in Figure 4c that the strategy choice of consumers also starts to fluctuate along with the fluctuation of the evolutionary path of logistics enterprises, and the magnitude of fluctuation is positively related to the strength of information disclosure.

(3) Impact of penalty intensity on evolutionary results

**Figure 5:**
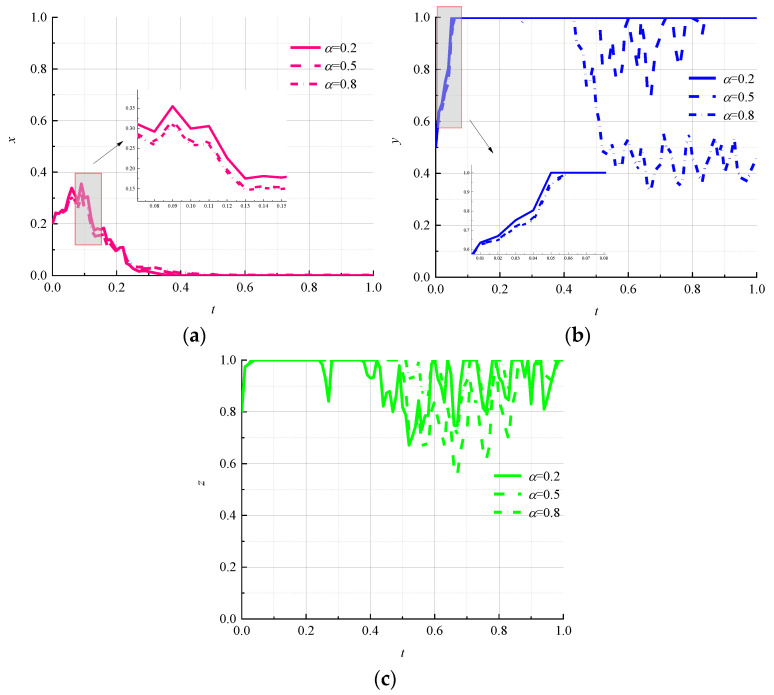
Evolutionary paths of each subject under different penalty strengths. (**a**) Environmental regulators; (**b**) logistics companies; (**c**) consumers.

Under strong government regulation, punitive policies will be introduced. Let α be 0.2, 0.5, and 0.8, corresponding to low strength, medium strength, and high strength, respectively, to explore the impact of the change in penalty intensity under strong regulation on the evolutionary path of each subject. The simulation results are shown in Figure 5.

Figure 5a shows that the environmental regulator evolves to the “strong regulation” strategy at the fastest rate under the high penalty; however, in general, the change in penalty level has minimal effect on the evolutionary path of the environmental regulator. Figure 5b shows that logistics companies shift to the “nonpromotion” strategy at the slowest rate under high-intensity penalties, and only under low-intensity penalties do they lock in the “nonpromotion” strategy and keep it stable; both medium- and high-intensity penalties can cause fluctuations in the evolutionary path of logistics companies. The fluctuation of logistics companies under high-intensity penalties is the largest and remains unstable, indicating that high-intensity penalties facilitate the choice of “promotion” strategy by logistics companies. From Figure 5c, it can be seen that the evolutionary path of consumers under different penalty levels fluctuates. Because the target of the penalty policy is the logistics enterprise, consumers do not have direct benefit loss; therefore, the fluctuation of consumers at this time is based on random disturbance in a random environment and does not show an obvious fluctuation pattern.

(4) Impact of subsidy intensity on evolutionary results

**Figure 6:**
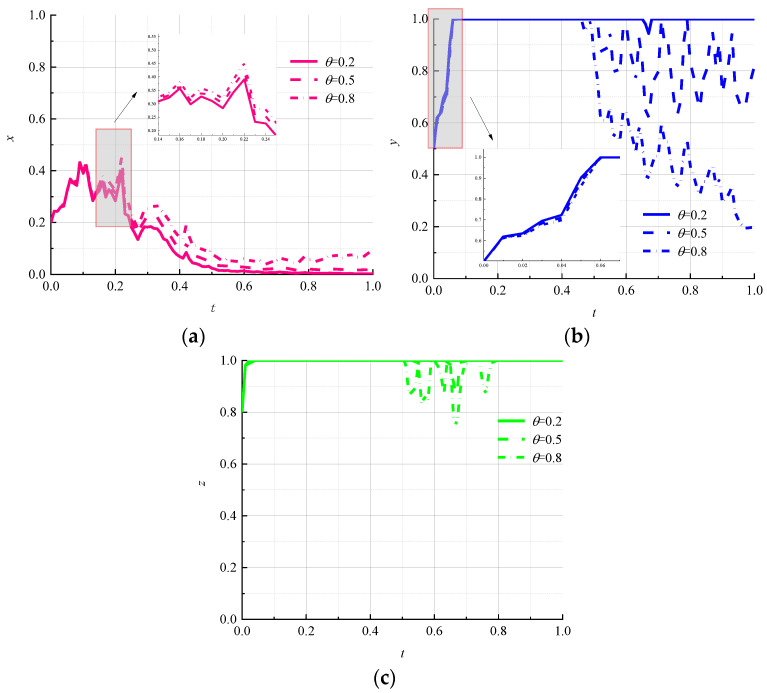
Evolutionary paths of each subject under different subsidy strengths. (**a**) Environmental regulators. (**b**) Logistics companies. (**c**) Consumers.

Whatever regulatory policy the government chooses, it will subsidize the “promotion” of logistics companies. Let θ be 0.2, 0.5, and 0.8, corresponding to low strength, medium strength, and high strength, respectively, to explore the evolutionary path of each subject under different subsidy strengths. The simulation results are shown in Figure 6.

It is clear that the rate of evolution of environmental regulators toward a “strong regulation” strategy is inversely related to the strength of subsidies, as shown in Figure 6a. Figure 6b shows that the evolutionary path of logistics companies gradually changes from a locked-in “nonpromotion” strategy to an unstable one with the increase in subsidies. The fluctuation of logistics enterprises is the largest under the high level of subsidies, and there is a tendency to choose the “promotion” strategy, indicating that the high level of subsidies has a positive effect on the promotion of circular packaging by logistics enterprises. In Figure 6c, similar to the penalty policy, the subsidies from environmental regulators to logistics firms have minimal effect on the evolutionary path of consumers; however, consumers also shift after the evolution of logistics firms’ strategies, and the magnitude of fluctuation is proportional to the intensity of the subsidies.

(5) Impact of the number by cycles on the evolutionary results

**Figure 7:**
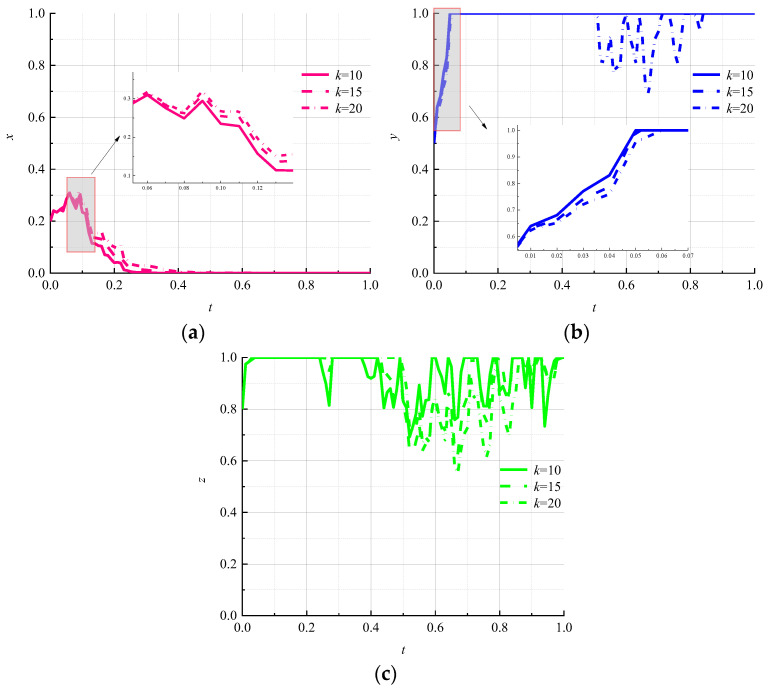
Evolutionary paths of each subject under different number of cycles. (**a**) Environmental regulators; (**b**) logistics companies; (**c**) consumers.

The number of cycles is an important indicator of the effectiveness of circular packaging. Let k be 10, 15, and 20, respectively, to study the effects of changes in the number of cycles on evolutionary paths. The simulation results are shown in Figure 7.

As the number of cycles increases, the rate of evolution of the environmental regulator toward the “strong regulation” strategy decreases slightly but does not affect its locking in the “strong regulation” strategy as shown in Figure 7a. It can be seen from Figure 7b that when the number of cycles reaches 20, the evolutionary path of logistics enterprises fluctuates, and the “nonpromotion” strategy cannot be locked, which indicates that the increase in the number of cycles can influence the choice of strategy of logistics enterprises. As shown in Figure 7c, the number of cycles is proportional to the fluctuation of the strategy choice of the consumers. When the number of cycles is 20, the fluctuation of the strategy choice of the consumers is the largest, and the “negative use” strategy cannot be locked, meaning that the increase in the number of cycles effectively increases the consumers’ active use of circular packaging.

(6) Impact of recycling incentives on evolutionary results

**Figure 8:**
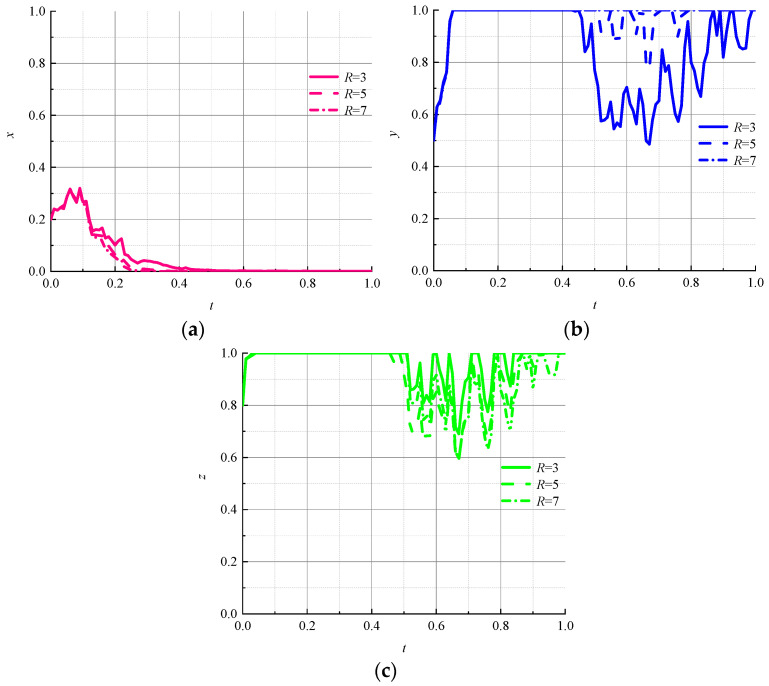
Evolutionary paths of each subject under different recycling incentives. (**a**) Environmental regulators; (**b**) logistics companies; (**c**) consumers.

Incentives given to consumers are an important means of motivating them to use circular packaging. Let R be 3, 5, and 7, respectively, to explore the evolutionary paths of game subjects under different recycling incentives. The simulation results are shown in Figure 8.

As shown in Figure 8a, the change in recycling incentive does not fluctuate significantly for the environmental regulator, and the rate of evolution toward a “strong regulation” strategy increases slightly with the increase in recycling incentive. The change in recycling incentives has a great impact on logistics companies and consumers (Figure 8b,c), and the magnitude of fluctuations in logistics companies is inversely proportional to the size of the recycling incentive. The magnitude of consumer volatility is proportional to the size of the recycling incentive; therefore, it can be seen that an increase in recycling incentives in a random-interference environment tends to make consumers shift toward the “active use” strategy, but a high recycling incentive can lock logistics companies into a “nonpromotion” strategy. How to set a reasonable recycling incentive is a question to that remains to be answered.

(7) Impact of random disturbance strength on evolutionary results

**Figure 9:**
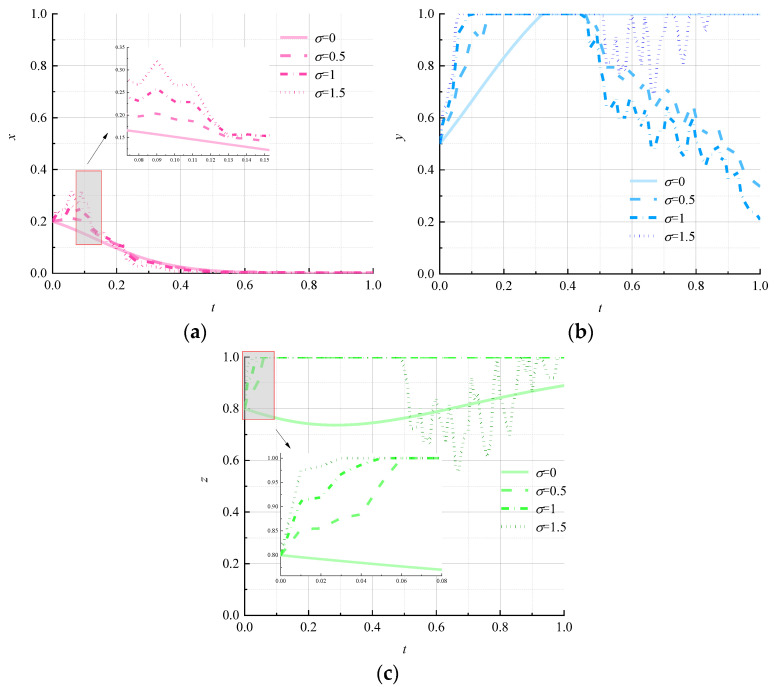
Evolutionary paths of each subject under different random-disturbance strengths. (**a**) Environmental regulators; (**b**) logistics companies; (**c**) consumers.

The strategic choices of environmental regulators in a stochastic environment may be influenced by public opinion, logistics companies may be speculative, and consumers may be influenced by irrational emotions. Let σ be 0, 0.5, 1, and 1.5, respectively, to study the evolutionary path of each subject under different disturbance intensities. The simulation results are shown in Figure 9.

Figure 9 shows that when the random disturbance intensity is 0, all three subjects of the game evolve with a smooth curve. The random interference increases the fluctuation of the strategy choice of the environmental regulator at the beginning of the game but accelerates the rate of evolution toward the “strong regulation” strategy at the later stage of the game (Figure 9a). The evolutionary path of logistics enterprises changes from locking in the “nonpromotion” strategy to the “promotion” strategy (Figure 9b) when the random interference strength is 0.5 and 1; the higher the strength of the random interference is, the faster is the rate of evolution toward the “promotion” strategy. Yet, when the random-interference strength continues to increase to 1.5, the logistics company will relock the “nonpromotion” strategy after a period of instability. This shows that moderate random interference can help logistics enterprises to promote circular packaging, whereas high-strength random interference can make the evolutionary path of the logistics enterprises fluctuate strongly but is not conducive in promoting circular packaging in logistics. Figure 9c shows that the increase in the strength of random interference accelerates the tendency of consumers to shift toward the “negative use” strategy at the beginning of the game; however, it can cause fluctuations in the choice of strategy of a consumer and a tendency to evolve toward the “positive use” strategy.

## 6. Conclusions and Policy Implications

The use of circular logistics packaging and the establishment of a recycling system is a complex issue. This study introduces random-interference coefficients based on an evolutionary game theory to build a game model for promoting circular logistics packaging, which consists of environmental regulators, logistics enterprises, and consumers, simulating the current situation of circular-logistics-packaging promotion and recycling in China to reveal the solutions to the current dilemma.

According to the numerical simulation, only the environmental regulator will lock in the “strong regulation” strategy, whereas logistics enterprises and consumers will lock in the “no promotion” and “negative use” strategies, respectively, in a random environment. To solve this dilemma, it is also necessary to develop appropriate penalties and information-disclosure mechanisms, strengthen the investment in circular-packaging technology, and research and develop circular logistics packaging that can be recycled many times in addition to strengthening the promotion of subsidies for logistics enterprises and consumer incentives for recycling. The main findings obtained from this study include the following: (1) environmental regulators have been actively promoting the use of circular packaging by enterprises and society. The higher the probability of the initial “strong regulation” strategy choice by environmental regulators is, the greater the fluctuation in strategy choice by logistics enterprises; the high level of regulation can drive logistics companies to choose “promotional” strategies. (2) In most cases, consumers’ strategic choices follow the fluctuations of logistics companies’ strategic choices. Even if environmental regulators choose a “weak regulation” strategy, if logistics companies are very willing to choose a “promotion” strategy at this time, the choice of strategy of the consumers will fluctuate greatly, and the probability of choosing “active use” increases significantly. (3) The high strength of punishment and information disclosure has a great impact on the strategy choice of logistics enterprises in addition to the subsidy policy, and the evolutionary path of logistics enterprises has not only fluctuated greatly but even tended to shift from locking in the “nonpromotion” strategy to locking in the “promotion” strategy. (4) In addition to the policy influence of environmental regulatory authorities, the amount of circular packaging recycling and the recycling incentives given by the logistics enterprises to consumers also affect the evolution path of the game system. The increase in the amount of circular-packaging recycling helps logistics enterprises shift toward the “promotion” strategy and consumers shift toward the “active use” strategy. The increase in the recycling incentive increases the magnitude of the fluctuation of consumers toward the “active use” strategy but encourages logistics enterprises to lock in the “nonpromotion” strategy; therefore, developing a reasonable incentive mechanism is an important issue in the promotion of circular packaging. (5) In a stochastic environment, an increase in the strength of the disturbance can intensify the fluctuation of the evolutionary paths of the game players; however, it is interesting to note that a moderate level of stochastic disturbance can help logistics companies evolve toward a “promotion” strategy. (6) Regardless of how the model parameters change, the Chinese government will lock in a “strong regulatory” strategy so that the strong regulatory strategy will be the dominant strategy of the Chinese government; the Chinese government will continue to promote the use of circular logistics packaging.

Based on the findings of this study, the following policy recommendations are made for the promotion and recycling of circular logistics packaging in China: (1) Given the considerable determination of the current government regulators to lock in “strong regulation,” it is recommended that environmental regulators increase subsidies, penalties, and information disclosures, as this will help establish a circular-logistics-packaging recycling system; (2) logistics companies should increase investment in the research and development of circular-packaging technology, increasing research and development costs in the short term but reducing packaging costs in the long term, as this will help in increasing the willingness of the consumers to choose “active use” strategies; (3) the recycling incentive for consumers should be increased, and the recycling incentive should be shared by the government and logistics enterprises, reducing the cost of logistics enterprises while helping consumers actively use circular packaging; (4) one of the major reasons for the current difficulties in circular-packaging promotion in China is the lack of consumer awareness of environmental protection, and consumers’ initial willingness to choose “active use” strategies is low. Therefore, the government and logistics companies need to expand the publicity of circular packaging and environmental protection.

## Figures and Tables

**Table 1:** Parameters symbol descriptions.

Parameters	Descriptions
$P_{1}$	Basic benefits for environmental regulators
$P_{2}$	Basic benefits for logistics companies
$P_{3}$	Basic benefits for consumers
M	Additional regulatory costs when environmental regulators choose a “strong regulation” strategy
B	Subsidies from environmental regulators for logistics companies choosing “promotion” strategies
θ	The intensity of subsidies from environmental regulators for logistics companies choosing “promotion” strategies
G	Environmental regulators fine logistics companies for choosing a “nonpromotion” strategy
α	The intensity of penalty from environmental regulators for logistics companies choosing “nonpromotion” strategies
$H_{1}$	Reputational damage of a logistics company’s choice of “nonpromotion” strategy when information is made public
$H_{2}$	Reputational benefits of a logistics company’s choice of “promotion” strategy when information is made public
β	The intensity of information disclosure by environmental regulators
$C_{1}$	Cost of ordinary logistics packaging
$C_{2}$	Cost of circular logistics packaging
k	The maximum number of cycles that can be made in a circular package
J	Cost of environmental governance for environmental regulators
E	Cost of developing circular packaging for logistics companies
W	Additional costs for logistics companies to promote circular packaging
R	Incentives for consumers to actively use circular packaging
T	The cost of time and physical effort for consumers to actively use circular packaging
I	The psychological loss of consumers after unsuccessful recycling
A	Potential losses for logistics companies

**Table 2:** Variable symbol descriptions.

Variable	Description
x	Probability of environmental regulators choosing a “weak regulation” strategy
y	The probability of logistics companies choosing the “nonpromotion” strategy
z	Probability of consumers choosing the “negative use” strategy

**Table 3:** Payoff matrix of government regulator, logistics enterprises, and consumers.

Strategic Choice	Environmental Regulators	Logistics Companies	Consumers
(*x*, *y*, *z*)	$P_{1}-J$	$P_{2}-C_{1}$	$P_{3}$
(1 − *x*, *y*, *z*)	$P_{1}+\alpha G+\beta S-M-J$	$P_{2}-C_{1}-\alpha G-\beta H_{1}$	$P_{3}$
(*x*, 1 − *y*, *z*)	$P_{1}-J-\theta B$	$P_{2}-C_{r}-E-W+\theta B$	$P_{3}$
(1 − *x*, 1 − *y*, *z*)	$P_{1}+\beta S-M-\theta B-J$	$P_{2}-C_{r}-E-W+\theta B+\beta H_{2}$	$P_{3}$
(*x*, *y*, 1 − *z*)	$P_{1}-J$	$P_{2}-C_{1}-A$	$P_{3}-I$
(1 − *x*, *y*, 1 − *z*)	$P_{1}+\alpha G+\beta S-M-J$	$P_{2}-C_{1}-\alpha G-\beta H_{1}-A$	$P_{3}-I$
(*x*, 1 − *y*, 1 − *z*)	$P_{1}-\theta B$	$P_{2}-C_{r}-E-W+\theta B-R$	$P_{3}+R-T$
(1 − *x*, 1 − *y*, 1−*z*)	$P_{1}+\beta S-M-\theta B$	$P_{2}-C_{r}-E-W+\theta B+\beta H_{2}-R$	$P_{3}+R-T$

**Table 4:** Values of parameters (Unit: million CNY).

Parameters	$C_{1}$	$C_{r}$	θB	αG	W	R	M	T	I	$\beta H_{1}$	$\beta H_{2}$	θ	α
**Value**	30	22.5	12	10	25	6	5	0.6	0.3	2	2	0.5	0.5
